# Immune Response of a Heterologous mRNA-1273 Second-Dose Immunization after a First Dose of ChadOx1 against SARS-CoV-2: A Cross-Sectional Study

**DOI:** 10.3390/vaccines10081241

**Published:** 2022-08-02

**Authors:** Beatrice Albanesi, Alessandro Godono, Rosanna Irene Comoretto, Elena Casabona, Giuliano Curoso, Massimiliano Victor Leone, Nicolò Milanesio, Ilenia Mirra, Giulia Montrucchio, Fabrizia Pittaluga, Rossana Cavallo, Marco Clari, Catalina Ciocan

**Affiliations:** 1 Department of Public Health and Pediatrics, University of Torino, 10126 Turin, Italy; beatrice.albanesi@unito.it (B.A.); alessandro.godono@unito.it (A.G.); rosannairene.comoretto@unito.it (R.I.C.); elena.casabona@unito.it (E.C.); giuliano.curoso@unito.it (G.C.); massimilianovictor.leone@unito.it (M.V.L.); nicolo.milanesio@unito.it (N.M.); ilenia.mirra@unito.it (I.M.); g.montrucchio@unito.it (G.M.); rossana.cavallo@unito.it (R.C.); catalina.ciocan@unito.it (C.C.); 2 Microbiology and Virology Unit, Città della Salute e della Scienza di Torino University Hospital, 10126 Turin, Italy; fpittaluga@cittadellasalute.to.it

**Keywords:** COVID-19, vaccines, heterologous, antibody response, spike protein, cross-sectional study

## Abstract

Heterologous vaccination regimens could contribute to broadening vaccination coverage. To date, there is little evidence on the effectiveness of a combination of adenoviral COVID-19 vaccines with a second dose of mRNA vaccines. This study aims to evaluate the antibody response to the SARS-CoV-2 spike protein 25 weeks after vaccination with mRNA-1273 after a first dose of ChAdOx1. A cross-sectional study was conducted collecting sociodemographic data, clinical characteristics, and serological data from among the general population. Antibody levels were expressed as binding antibody units (BAU) per mL (cutoff = 33.8 BAU/mL). Linear regression models were used to assess the relationship between the subjects’ characteristics and anti-SARS-CoV-2 antibody levels. A total of 229 participants were followed up after a median time of 173 days. The overall anti-SARS-CoV-2 IgG antibody titer was 729.0 BAU/mL. The multivariable analysis showed that the only factor associated with anti-SARS-CoV-2 IgG levels was the BMI (*p* = 0.007), with decreases within the healthy range weight and increases in under- or overweight people. Our results support the use of heterologous COVID-19 vaccination regimens, as they can guarantee a sustained immune antibody response. More studies are needed to understand the link between BMI and body composition and the immune response to COVID-19 vaccinations.

## 1. Introduction

Today, the world is still struggling to recover from uninterrupted waves of coronavirus disease 19 (COVID-19) caused by severe acute respiratory syndrome coronavirus 2 (SARS-CoV-2) [1,2]. In addition to extensive lockdowns and hygiene control measures implemented globally, the most recognized and effective way to prevent COVID-19 infection, hospitalization, and associated deaths has been mass vaccination [1].

Currently, six COVID-19 vaccines are authorized in the European Union by the European Medicines Agency (EMA)—two mRNA vaccines: BNT162b2 (Comirnaty^®^, Pfizer-BioNTech, New York, NY, USA) and mRNA-1273 (Spikevax^®^, Moderna, Cambridge, MA, USA); two viral vector vaccines: ChAdOx1 nCoV-19 (Vaxzevria^®^, Astrazeneca, Cambridge, UK) and Ad26.COV2.S (Janssen^®^, Beerse, Belgium); one protein-based vaccine: NVX-CoV2373 (Novavax^®^, Gaithersburg, MD, USA); and one inactivated, adjuvant vaccine: (Valneva^®^, Saint-Herblain, France) [3].

Despite an exceptional promptness in the development of the COVID-19 vaccine, vaccination programs faced several barriers [4]. Production delays, logistical challenges, vaccine-induced immune thrombocytopenia (VITT) after immunization with adenoviral vector vaccines, and highly transmissible and resistant variants of SARS-CoV-2 increased the need for adapting vaccination regimens combining different vaccines. 

Generally, experimental evidence has suggested that heterologous vaccination improves the immune response [5]; indeed, heterologous vaccination has been applied in various vaccination programs against other viruses (e.g., Ebola) [6]. Focusing on COVID-19, several studies investigated the immunogenicity and reactogenicity of such regimens, principally combining the adenoviral vaccine ChAdOx1 with the mRNA vaccine BNT162b2 [7]. 

The literature has consistently reported a greater effect of heterologous vaccination. The study by Liu et al. [8] showed that a heterologous regimen with a 4-week prime–boost interval, combining the ChAdOx1 nCoV-19 and BNT162b2 vaccines, stimulated a higher immune response than a homologous regimen (ChAdOx1/ChAdOx1). The study reported significantly higher neutralizing antibody titers and higher T-cell responses. A significant increase in the frequency of memory B cells (RBD) and a stronger T-cell response was observed after 4-week heterologous ChAdOx1 and BNT162b2 vaccination by Pozzetto et al. [8]. Moreover, this study showed a stronger neutralizing activity regardless of the SARS-CoV-2 beta variant. A significant increase in antibody titers was also observed after the 8-week study by Gross et al. [9], and after the 4-week study by Westrop et al. [10], where antibodies also remained significantly higher in heterologous-vaccinated individuals with ChAdOx1 and BNT162b2 after 12 weeks, as compared to BNT162b2/BNT162b2 or ChAdOx1/ChAdOx1. Hillus et al. [11] showed that heterologous vaccination (ChAdOx1/BNT162b2) with a vaccine interval of 10–12 weeks is at least as immunogenic as homologous vaccination with BNT162b2 with a 3-week prime–boost interval. The CombivacS study [12] demonstrated that a second dose of BNT162b2 administered 8–12 weeks after a primer of ChAdOx1 nCoV-19 generated a robust immune response; furthermore, the proportion of subjects who reached an antibody titer of over 75 AU/mL was higher after 15 weeks in the heterologous ChAdOx1 and BNT162b2 group than in the homologous group (BNT162b2/BNT162b2) [13]. 

Less evidence has been produced on the immunogenicity of heterologous vaccination combining the adenoviral vaccine ChAdOx1 with the mRNA-1273 vaccine [14,15,16,17]. Only one study tested the combination of ChAdOx1 with mRNA-1273, showing that SARS-CoV-2 spike protein (S) and RBD-specific IgG levels were significantly higher [14]. Gram et al. [15], in a test of 2-week heterologous mRNA vaccinations (BNT162b2 or mRNA-1273), obtained 92% vaccination effectiveness (VE) in heterologous-vaccinated individuals versus a VE of 58% in the homologous group given ChAdOx1/ChAdOx1. Cellular and serological immune responses were significantly stronger with mRNA vaccines, including against variants of concern (VOCs; *p* < 0.005), in the 15-week study by Fabricius et al. [16], while an increase in spike-specific IgG and spike-specific T-cell levels was observed in the 11-week study by Schmidt et al. [17] after heterologous vaccination. 

This evidence indicates that a heterologous prime–boost regimen could be used due to the high elicitation and reactogenicity of the immune response, similar to homologous regimens [18]. However, these findings should be interpreted with caution due to the lack of long-term follow-up on vaccination safety profiles, along with their immunological outputs and clinical impacts [19]. Furthermore, although the mRNA-1273 vaccine has been largely used against COVID-19, few studies have investigated the antibody response after a second boost of heterologous vaccination with mRNA-1273. Lastly, all studies investigated small sample sizes with short follow-ups.

Therefore, this study has set two main objectives: (i) to test the antibody response to the SARS-CoV-2 spike protein 25 weeks after a heterologous regimen with vaccination with mRNA-1273 after a first dose of ChAdOx1; and (ii) to evaluate the associated characteristics of this antibody response.

## 2. Materials and Methods

A cross-sectional study was conducted.

### 2.1. Participants and Setting

To be included in the study, individuals had to (i) be between 18 and 60 years of age; (ii) have received a first dose of the ChAdOx1 nCoV-19 vaccine (Vaxzevria^®^) between 27 March and 8 April 2021, and a second dose of the mRNA-1273 vaccine (Spikevax^®^) between 16 June and 1 July 2021; and (iii) not be infected by SARS-CoV-2 between the 2 doses. Participants were subjected to a blood test at 25 weeks after the second dose (from 15 November to 31 December 2021).

The site for the vaccination was in Northwest Italy (Piedmont), operated by the University of Torino in collaboration with the local health authority (ASL TO 1). Blood samples were collected and processed at the local hospital (AOU Città della Salute e della Scienza di Torino).

### 2.2. Data Collection

An email requesting interest in participation and containing a study information sheet was sent to the possible participants. A questionnaire was created according to the literature and administered before blood sampling. The data on sociodemographic characteristics (i.e., sex, age) and relevant clinical characteristics (i.e., body mass index (BMI), presence of coagulopathy, allergies, illness- or therapy-related immunosuppression, relevant changes in health status in the period between the first and the second dose and, finally, SARS-CoV-2 infection after the second dose of the vaccine) were collected. 

### 2.3. Ethical Consideration

The study was carried out according to the guidelines of the Declaration of Helsinki, and was approved by the Bioethics Committee of the University of Torino (Approval No. 0596391). All study participants voluntarily adhered to the study. Before participating they were informed about the study’s rationale, aims, and anonymity. Participants did not receive any incentive to participate in the study. Only the research team had access to the database, and the data were collected and treated anonymously.

### 2.4. Blood Sample Analysis

#### 2.4.1. Serological Assay

Serological data on serum specimens were collected using the LIAISON^®^ SARS-CoV-2 TrimericS IgG indirect chemiluminescent immunoassay (CLIA) (DiaSorin, Saluggia, Italy), following the manufacturer’s instructions, and using the LIAISON^®^ XL analyzer. This assay uses a recombinant trimeric spike glycoprotein as a capture antigen coated on magnetic particles (solid phase) and murine monoclonal antibodies to human IgG linked to an isoluminol derivative (conjugate). Antibody levels were calculated by the analyzer and expressed as binding antibody units (BAU) per mL, with a cutoff value for positivity of 33.8 BAU/mL, allowing for a qualitative grading of the results: <33.8 BAU/mL considered as negative; ≥33.8 BAU/mL as positive.

According to the manufacturer, the sensitivity and specificity of the LIAISON^®^ SARS-CoV-2 TrimericS IgG test are 98.7% and 99.5%, respectively, and the positive agreement with the plaque reduction neutralization test (considered the gold standard for detecting and measuring antibodies that can neutralize viruses) is 100%. 

#### 2.4.2. Molecular Assay

SARS-CoV-2 RNA was obtained from upper respiratory specimens (nasopharyngeal swab) by a commercial molecular test—the AptimaTM SARS-CoV-2 Assay with the PantherTM Fusion System (Hologic, Rome, Italy)—following the manufacturer’s instructions. This assay received emergency use authorization from the Food and Drug Administration. Briefly, the assay combines the technologies of target capture, transcription-mediated amplification, and dual kinetic assay, and detects two conserved regions of the ORF1ab gene. Quantitative results were determined by a cutoff based on the total relative light units and the type of kinetic curve.

### 2.5. Statistical Analysis

The descriptive analysis of the study sample was reported using the median and interquartile range (IQR) for continuous variables, and using frequencies and relative percentages for categorical variables. The study sample was stratified at baseline according to sex. Unadjusted differences between men and women were tested using Wilcoxon or chi-squared tests whenever appropriate, depending on the variable analyzed. The association between main sample characteristics and anti-SARS-CoV-2 IgG levels (outcome) was estimated using univariable linear models. Furthermore, the effects of relevant confounders on the outcome were considered by estimating multivariable linear models. Variables to be included were selected according to clinical judgment. The nonlinear effects of the covariates were estimated using restricted cubic splines, and their significance was estimated using a log-likelihood ratio test. The goodness of fit was evaluated using Somers’ D and R^2^ on a set of bootstrapped (B = 10,000) resamples. Statistical significance was established with a two-sided *p*-value < 0.05. The Benjamini–Hochberg correction was applied to control the false discovery rate due to multiple testing. The analysis was performed using the statistical software R (version 4.1.1) and the RMS libraries.

## 3. Results

In total, 1089 individuals were vaccinated with ChAdOx1 nCoV-19 as the first dose and mRNA-1273 as the second dose. Of these, 701 were excluded for the following reasons: 4 had already received a third dose, and 697 were uncontactable. Of the 388 contacted individuals, 89 declined to participate.

The study sample of 299 subjects was followed up and received the heterologous second dose after a median time of 173 (IQR: 171–177) days. The median age of patients was 35.0 years, and most of them were males (n = 161, 53.8%). Males had a lower BMI than females (21.4 vs. 23.9, *p* < 0.001). Twenty-seven percent of patients reported being affected by allergies, with a higher rate in the female sex. The overall anti-SARS-CoV-2 IgG was 729.0 BAU/mL (IQR: 455.0–1205.0 BAU/mL). Males had higher median levels of anti-SARS-CoV-2 IgG antibody titer, but this difference was not statistically significant (*p* = 0.318) (Table 1).

**Figure 1:**
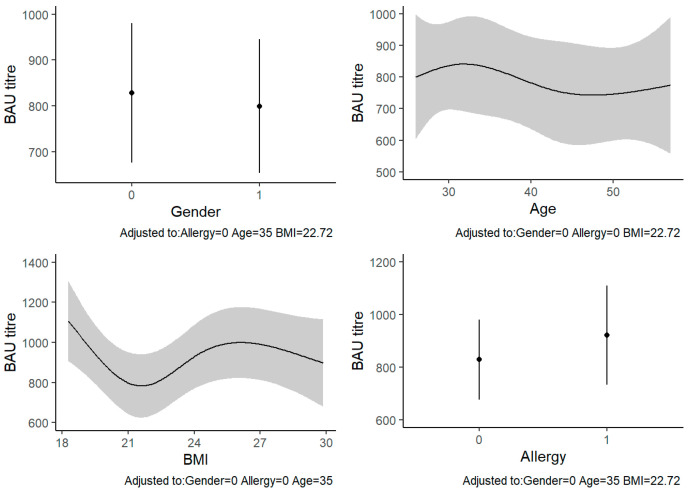
Multivariable linear regression between BAU titers (BAU/mL) and sample characteristics: The four plots report the predicted values of BAU titer for each model’s covariates, adjusted for the other variables. Abbreviations: BAU, binding antibody units; BMI, body mass index.

The univariable analyses showed that the only characteristic significantly associated with anti-SARS-CoV-2 IgG levels was BMI (*p* = 0.007) (Table 2). In the multivariable analysis, BMI remained significantly associated with the outcome (β = 172.3, 95% CI: 2.9–341.6, *p* = 0.007). In particular, the anti-SARS-CoV-2 IgG levels seemed to decrease in people with a healthy weight range and increase in underweight or overweight people. At a BMI around 30, the IgG levels decreased again. The other variables were not significantly associated with anti-SARS-CoV-2 IgG levels (Table 3, Figure 1).

When analyzing the sample characteristics according to sex, BMI remained significantly associated with anti-SARS-CoV-2 IgG levels only in females (β = 277.0, 95% CI: 37.3–516.7, *p* = 0.045), with the same trend as described previously. No statistically significant association for the other variables was found (Table 4).

## 4. Discussion

This study evaluated antibody responses to vaccination with a heterologous SARS-CoV-2 regimen at 25 weeks in subjects who received a first dose of ChAdOx1 followed by mRNA-1273, along with the characteristics of associated individuals. 

The overall response in SARS-CoV-2 IgG after a median of 173 days was 729.0 BAU/mL. Compared to the values reported in another recent study [20], the mean antibody titer in our population appeared to be significantly higher. In particular, the study by Guiomar et al. [20] analyzed the immune responses of healthcare workers by collecting blood samples before vaccination, at the second dose of vaccine uptake, and 25–70 days and 150–210 days (month 4) after the second dose. Participants were vaccinated with a homologous regimen, mainly with BNT162b2. The mean antibody titer was 1250.1 BAU/mL at month 3 after 25–70 days from the second dose, and it reduced after 150–210 days, falling to 152 BAU/mL. Likewise, a study conducted in the same geographical area in a similar population showed IgG values of 990 BAU/mL approximately three months after the second dose. The results by Costa et al. [21], despite the shorter time between the second dose and the IgG titer, are the most comparable due to their reduced sociodemographic variability. Furthermore, Schmidt et al. [17] evaluated the antibody responses of adult individuals after 14 days, and observed the ability of heterologous regimens (BNT162b2 and mRNA-1273) to induce a stronger immunological response compared to homologous regimens. Specific IgG levels after heterologous vaccination and homologous mRNA vaccination were similar (3630 and 4932 BAU mL^−1^, respectively), while levels after homologous vector vaccination were significantly lower (404 BAU mL^−1^, *p* < 0.0001). This difference was also observed for neutralizing antibodies, which were significantly lower in homologous vector groups. Raposo et al. [22], in their evaluation of heterologous prime–boost vaccination, concluded that the BNT162b vaccine was efficient in maintaining an antibody response in both previously vaccinated ChAdOx1 nCoV-19 recipients, and as a prime vaccine in COVID-19-infected receivers. In particular, the basal BAU/mL seemed to increase after the second dose of the heterologous vaccine, with a peak at 14 days (>2000 BAU/mL) followed by a decrease after 21 days. Although the time intervals considered in these studies were not identical to those of our study, it could be possible that the longer interval in our study could have influenced the decline in antibody titers.

One of the main results of our study is that BMI appears to be the only individual characteristic significantly associated with anti-SARS-CoV-2 IgG levels. Our analyses showed that antibody levels were lower in healthy-weight people than in underweight or overweight people. The importance of BMI and weight levels in the immune response to vaccines is a well-known phenomenon [23], and has already been investigated in other infectious diseases [24]. For example, previous studies have reported that obesity contributes to lower immune responses to seasonal influenza vaccines, increasing the risk of developing more severe influenza symptoms [25]. Similarly, a high BMI was the third most significant factor for poor vaccine-induced anti-HBs titers [26], and a slower antibody response to hepatitis A vaccination was observed in overweight individuals compared to normal-weight subjects [26,27]. Elevated BMI might interfere with the individual’s ability to mount an effective immune response to vaccination or infection, due to high levels of body fat and the increased production of leptin—a pro-inflammatory hormone with important immunological functions [28].

On the other hand, underweight individuals are also subject to altered cytokine production. Therefore, both obesity and malnutrition led to a change in the immune response. To date, emerging evidence shows that many nutritionally regulated hormones mediate immune cell metabolism [29]. Our results confirm that nutritional status could affect the immunological function, but more research is needed to understand the precise mechanisms underlying immunity mediated by over- or undernutrition.

Several studies have already investigated the relationship between BMI and immune response to SARS-CoV-2 vaccination. Recently, Bates et al. [30] highlighted that in a cohort of 126 individuals vaccinated with two doses of BNT162b2, BMI did not significantly affect vaccine-specific antibody titers [29]. These results are consistent with those of Pellini et al. [31]. Conversely, Malavazos et al. [32] showed that an elevated BMI could reduce antibody levels in immunized individuals with BNT162b2 about 100 days after vaccine-induced immunization. Similarly, another recent study has reported a negative effect of high BMI levels on antibody responses among males, but not females [33]. The heterogeneity of these results could be explained by a faster antibody decay phase in individuals with high BMI. Furthermore, the immunological response could be related to the type of vaccine proposed. The relationship between sex, BMI, and antibody titers has been studied by Kuo et al. [34], who supported the hypothesis that age and BMI may have sex-specific effects on antibody responses to the influenza A virus vaccine. In that study, the decline in pre-vaccination H3N2 titers with increasing BMI was higher in female compared to male healthcare workers. Furthermore, in the study by Pellini et al. [35], which tested a homologous vaccination at 7 and 21 days in a sample of healthcare workers, it was found that antibody titers were higher in young and female individuals than in men and older subjects. In females, a strong correlation between BMI classes and antibody titers was observed; the humoral response was more efficient in under- and over-weight subjects compared to normal-weight and obese ones (*p* < 0.0001). Despite this evidence, the exact mechanism behind the association between BMI and IgG levels is still not fully understood. In particular, as this is a nonlinear association, a complete understanding of BMI and other related factors is even more difficult, and merits further investigation. 

Our study has several strengths. Firstly, it is one of the few studies to evaluate the immune response in a heterologous regimen with mRNA-1273 vaccination after a first dose of ChAdOx1. To our knowledge, no other studies have investigated IgG levels 6 months after immunization with this type of vaccination regimen. Furthermore, the size of our sample is quite large compared to other similar studies; this aspect could ensure a good representativeness of our sample. A limitation of our study design is the absence of antibody values at baseline, which prevents the possibility of assessing the trend of IgG titers over time and defining proper causal links. In addition, we did not recruit a control group, but descriptive comparisons were still possible due to the broad scientific literature related to other vaccination regimens.

## 5. Conclusions

This study highlights the medium-term immunogenicity of the heterologous COVID-19 vaccination regimen, providing data for up to 25 weeks after the second dose, supporting the current guidance to increase the use of mRNA vaccines after an adenoviral-vectored prime. Our results show that BMI is a factor significantly associated with SARS-CoV-2 vaccine response. However, unlike other studies, we found that IgG levels increased in under- or overweight people. More studies on the effects of BMI and body composition are needed to explore the relationship between adiposity and the immune response of COVID-19.

## Figures and Tables

**Table 1:** Baseline characteristics based on sex.

Characteristics				*p*-Value
	Males (n = 161)	Females (n = 138)	All (n = 299)	
Age (years; median, IQR)	36.0 (30.0–47.0)	35.0 (29.2–47.0)	35.0 (30.0–47.0)	0.712
BMI (kg/m^2^; median, IQR)	21.5 (19.5–24.0)	23.9 (22.2–25.7)	22.7 (20.7–25.1)	<0.001
<25 (n, %)	130 (81)	93 (67)	223 (75)	0.009
≥25 (n, %)	31 (19)	45 (33)	76 (25)	
Allergies (yes; n, %)	41 (25)	40 (29)	81 (27)	0.513
Days from 2nd dose (median, IQR)	173 (171–176)	173 (171–178)	173 (171–177)	0.962
Anti-SARS-CoV-2 IgG(BAU/mL; median, IQR)	773.0 (508.0–1190.0)	683.5 (411.0–1207.5)	729.0 (455.0–1205.0)	0.318

Abbreviations: BMI, body mass index; IQR, interquartile range.

**Table 2:** Univariable analysis of the association between anti-SARS-CoV-2 titers and sample characteristics (all participants).

Factors	β (SE)	95% CI	*p*-Value
Sex	−44.5 (64.1)	−170.6–81.7	0.488
Age *	−112.7 (88.5)	−287.0–61.5	0.649
BMI *	137.6 (87.0)	−33.5–308.7	0.007
Allergies	97.2 (71.7)	−44.0–238.3	0.176
Days from 2nd dose	11.8 (45.9)	−78.5–102.1	0.798

* Nonlinear analysis. Abbreviations: SE, standard error; CI, confidence interval; BMI, body mass index.

**Table 3:** Multivariable analysis of the association between anti-SARS-CoV-2 titers and sample characteristics (all participants).

Factors	β (SE)	95% CI	*p*-Value
Sex	−29.3 (69.2)	−165.5–106.8	0.672
Age *	−93.4 (88.0)	−266.5–79.7	0.789
BMI *	172.3 (86.0)	2.9–341.6	0.007
Allergies	93.3 (71.7)	−47.9–234.5	0.194

* Nonlinear analysis. Abbreviations: SE, standard error; CI, confidence interval; BMI, body mass index.

**Table 4:** Multivariable analysis of the association between anti-SARS-CoV-2 IgG and sample characteristics by sex.

Factors	Males			Females		
	β (SE)	95% CI	*p*-Value	β (SE)	95% CI	*p*-Value
Age *	−84.1 (116.6)	−314.5–146.3	0.630	−97.9 (129.4)	−353.9–158.1	0.671
BMI *	33.9 (114.4)	−192.1–259.9	0.079	277.0 (121.2)	37.3–516.7	0.045
Allergies	130.6 (100.4)	−67.8–339.0	0.196	48.1 (106.3)	−162.1–254.4	0.651

* Nonlinear analysis. Abbreviations: SE, standard error; CI, confidence interval; BMI, body mass index.

## Data Availability

The data are not available to the public without the consent of the corresponding authors (R.I.C. and M.C.).
